# Supplementary material for: A Likelihood Approach for Real-Time Calibration of Stochastic Compartmental Epidemic Models
Source: PLoS Comput Biol. 2017 Jan 17;13(1):e1005257. doi: 10.1371/journal.pcbi.1005257 (PMC5240920; doi:10.1371/journal.pcbi.1005257)
Supplement: S1 File — (TAR.GZ) [file pcbi.1005257.s014.tar.gz › HSPH_Online-SI-Revision/IPoi09/n10-severe/n10-severe_BENCH_table8.pdf]

1	R0	{1.68952, 11.8402}	{2.46477, 18.9032}	{{2.46477, 18.9032}, {2.46477, 18.9032}}	{45.8858, 59.6527}	inf	198.	533.85	{220., 884.}	170.21	0.362818	newly	99.	178.65	{71., 309.}	92.0034	0.234557	Reff	0.571396	0.363085	{0.28616, 0.416546}	44.8945	ar	6872.	8883.57	{8780., 8947.}	29.2719
2	R0	{1.71739, 9.62661}	{2.64373, 19.7855}	{{2.64373, 19.7855}, {2.64373, 19.7855}}	{53.9388, 105.529}	inf	184.	925.825	{470., 1599.}	403.166	0.650657	newly	116.	296.571	{149., 508.}	155.665	0.35322	Reff	0.569658	0.356278	{0.279442, 0.45525}	40.3014	ar	6874.	9088.49	{9032., 9142.}	32.2155
3	R0	{1.48936, 6.23975}	{2.60953, 15.4938}	{{2.60786, 15.4074}, {2.60786, 15.5255}}	{75.2115, 148.308}	inf	38.	383.344	{158., 844.}	908.799	0.907076	newly	40.	149.922	{65., 328.}	274.805	0.478509	Reff	0.649957	0.285372	{0.238358, 0.337979}	56.0938	ar	5666.	9052.41	{8968., 9145.}	59.7671
4	R0	{1.62394, 7.51135}	{2.42205, 19.4475}	{{2.42205, 19.4475}, {2.42205, 19.4475}}	{49.1465, 158.908}	inf	86.	577.621	{232., 1053.}	571.652	0.736095	newly	66.	188.69	{71., 335.}	188.61	0.371156	Reff	0.601507	0.388576	{0.30203, 0.426523}	44.7867	ar	6417.	8811.98	{8743., 8883.}	37.3225
5	R0	{1.53632, 8.57652}	{2.42205, 19.4475}	{{2.42205, 19.4475}, {2.42205, 19.4475}}	{57.6527, 126.753}	inf	148.	577.621	{232., 1053.}	290.424	0.500946	newly	112.	188.69	{71., 335.}	89.5628	0.243314	Reff	0.610687	0.388576	{0.30203, 0.426523}	45.4611	ar	6260.	8811.98	{8743., 8883.}	40.7665
6	R0	{1.47166, 5.7434}	{2.43251, 19.1951}	{{2.42205, 19.0531}, {2.43839, 19.4475}}	{65.2901, 234.211}	inf	52.	746.848	{332., 1337.}	1336.25	1.09043	newly	51.	243.5	{98., 458.}	377.451	0.60531	Reff	0.657979	0.400982	{0.310651, 0.517426}	43.9771	ar	5650.	8833.35	{8743., 8921.}	56.3425
7	R0	{1.48179, 8.87369}	{2.37866, 17.4909}	{{2.36153, 17.2664}, {2.50427, 19.1373}}	{60.526, 97.1097}	inf	96.	451.339	{159., 985.}	370.145	0.556855	newly	76.	164.102	{51., 361.}	127.877	0.27322	Reff	0.721187	0.371919	{0.302512, 0.407128}	51.9506	ar	5233.	8743.73	{8617., 8889.}	67.0883
8	R0	{1.53669, 6.47807}	{2.86656, 18.9813}	{{2.86656, 18.9813}, {2.86656, 18.9813}}	{86.5412, 193.009}	inf	51.	577.59	{311., 1235.}	1032.53	0.984696	newly	34.	186.656	{92., 373.}	448.987	0.66712	Reff	0.629736	0.252036	{0.213272, 0.291242}	59.9774	ar	5934.	9306.52	{9263., 9354.}	56.8339
9	R0	{1.57227, 6.20989}	{2.96947, 15.963}	{{2.96947, 15.963}, {2.96947, 15.963}}	{88.8651, 157.058}	inf	66.	513.	{252., 822.}	677.273	0.817262	newly	57.	190.355	{91., 277.}	233.956	0.450048	Reff	0.625135	0.227073	{0.188561, 0.260126}	63.6761	ar	6136.	9383.39	{9320., 9450.}	52.9235
10	R0	{1.54936, 6.50938}	{2.95209, 14.037}	{{2.95209, 14.037}, {2.95209, 14.037}}	{90.5361, 115.643}	inf	32.	214.806	{110., 362.}	571.27	0.760742	newly	31.	89.3871	{42., 160.}	189.074	0.390402	Reff	0.582559	0.202342	{0.181554, 0.223473}	65.2667	ar	6275.	9365.42	{9314., 9419.}	49.2497
11	R0	{1.42179, 3.18601}	{2.45839, 11.6881}	{{2.45839, 11.6881}, {2.45839, 11.6881}}	{72.9081, 266.857}	inf	5.	154.098	{56., 232.}	2981.97	1.37123	newly	7.	75.4098	{26., 119.}	977.283	0.904615	Reff	0.665398	0.293532	{0.272144, 0.316886}	55.8863	ar	5322.	8870.92	{8797., 8938.}	66.6839
12	R0	{1.48035, 6.46183}	{2.69392, 14.8759}	{{2.688, 14.8279}, {2.688, 14.8279}}	{81.9788, 130.212}	inf	58.	440.468	{185., 696.}	659.427	0.794301	newly	54.	175.984	{74., 270.}	226.374	0.424141	Reff	0.636995	0.274131	{0.234841, 0.308851}	56.9649	ar	5793.	9145.48	{9083., 9222.}	57.8713
13	R0	{1.75755, 8.53854}	{2.85956, 19.8999}	{{2.85956, 19.8999}, {2.85956, 19.8999}}	{62.7015, 133.06}	inf	105.	591.279	{299., 959.}	463.123	0.683915	newly	83.	185.131	{91., 297.}	125.025	0.289421	Reff	0.574719	0.255452	{0.215325, 0.294249}	56.0529	ar	6832.	9302.69	{9253., 9345.}	36.1635
14	R0	{1.47355, 4.89451}	{2.66419, 14.2056}	{{2.66419, 14.2056}, {2.66419, 14.2056}}	{80.8008, 190.235}	inf	18.	238.419	{111., 346.}	1224.55	1.03177	newly	20.	97.7903	{46., 151.}	388.952	0.597875	Reff	0.635542	0.256265	{0.227255, 0.286933}	59.6777	ar	5710.	9121.95	{9067., 9185.}	59.754
15	R0	{1.62071, 5.98665}	{2.84265, 10.712}	{{2.83858, 10.6973}, {2.83858, 10.6973}}	{75.3953, 78.9312}	inf	12.	75.5238	{34., 106.}	529.365	0.723549	newly	11.	38.873	{17., 56.}	253.68	0.467009	Reff	0.547962	0.207567	{0.190469, 0.222829}	62.1203	ar	6635.	9288.76	{9235., 9352.}	39.9964
16	R0	{1.67268, 7.7256}	{2.96947, 15.963}	{{2.96947, 15.963}, {2.96947, 15.963}}	{77.5277, 106.625}	inf	38.	187.629	{82., 324.}	393.761	0.606592	newly	29.	69.9194	{30., 104.}	144.105	0.305305	Reff	0.519367	0.197168	{0.174902, 0.219147}	62.0369	ar	6947.	9383.39	{9320., 9450.}	35.0711
17	R0	{1.67805, 11.1417}	{2.96804, 18.8836}	{{2.96978, 18.8857}, {2.96978, 18.8857}}	{76.8741, 69.4862}	inf	310.	648.95	{283., 1191.}	113.059	0.263643	newly	183.	210.183	{86., 376.}	52.0674	0.189708	Reff	0.643364	0.241902	{0.195412, 0.296716}	62.6183	ar	6625.	9382.78	{9331., 9440.}	41.6269
18	R0	{1.66743, 9.99046}	{2.85956, 19.8999}	{{2.85956, 19.8999}, {2.85956, 19.8999}}	{71.4951, 99.189}	inf	216.	748.328	{389., 1207.}	246.448	0.480862	newly	133.	234.131	{113., 374.}	82.5958	0.211704	Reff	0.574763	0.277495	{0.223332, 0.318841}	53.389	ar	6899.	9302.69	{9253., 9345.}	34.8411
19	R0	{1.70787, 9.85478}	{2.42205, 19.4475}	{{2.42205, 19.4475}, {2.42205, 19.4475}}	{41.817, 97.3408}	inf	121.	690.828	{292., 1241.}	470.932	0.681269	newly	78.	229.	{90., 409.}	195.314	0.390794	Reff	0.56428	0.416622	{0.311233, 0.470362}	40.7583	ar	6867.	8811.98	{8743., 8883.}	28.3236
20	R0	{1.53809, 10.5848}	{2.30371, 19.6625}	{{2.30371, 19.6625}, {2.30371, 19.6625}}	{49.7773, 85.7617}	inf	283.	677.909	{345., 1142.}	140.713	0.345175	newly	160.	218.345	{119., 373.}	48.8523	0.159831	Reff	0.669684	0.462078	{0.354771, 0.497141}	45.3798	ar	6108.	8636.65	{8547., 8714.}	41.3991
21	R0	{1.59925, 6.74479}	{2.95369, 14.2038}	{{2.95209, 14.037}, {2.96307, 14.3219}}	{84.6925, 110.589}	inf	59.	228.882	{107., 417.}	287.935	0.527259	newly	39.	93.1447	{41., 174.}	140.992	0.323997	Reff	0.593482	0.201875	{0.183915, 0.227016}	65.9846	ar	6411.	9370.93	{9319., 9424.}	46.1696
22	R0	{1.38779, 4.40977}	{2.39302, 13.3816}	{{2.36011, 13.1698}, {2.42221, 13.5694}}	{72.4341, 203.453}	inf	49.	390.779	{131., 949.}	697.509	0.779006	newly	54.	173.338	{57., 386.}	222.358	0.383007	Reff	0.682793	0.340997	{0.292603, 0.39046}	51.073	ar	5154.	8785.59	{8680., 8896.}	70.4615
23	R0	{1.53657, 7.50352}	{2.79411, 17.615}	{{2.75274, 17.3228}, {2.96978, 18.8857}}	{81.8406, 134.757}	inf	141.	443.282	{172., 865.}	214.884	0.389137	newly	110.	154.113	{56., 312.}	71.9846	0.211501	Reff	0.661647	0.253694	{0.208182, 0.282431}	61.844	ar	5928.	9246.31	{9145., 9391.}	55.9769
24	R0	{1.71131, 6.24526}	{2.8833, 17.1847}	{{2.8833, 17.1847}, {2.8833, 17.1847}}	{68.485, 175.164}	inf	47.	522.951	{248., 947.}	1012.66	0.979248	newly	36.	183.115	{87., 326.}	408.652	0.639003	Reff	0.538036	0.243473	{0.211058, 0.270742}	54.7477	ar	6924.	9313.56	{9263., 9361.}	34.5112
25	R0	{1.4412, 3.07729}	{2.47628, 10.2848}	{{2.47628, 10.2848}, {2.47628, 10.2848}}	{71.8207, 234.216}	inf	2.	118.919	{43., 185.}	5845.97	1.65627	newly	2.	63.9355	{22., 91.}	3096.77	1.38826	Reff	0.603863	0.284341	{0.262486, 0.313497}	52.913	ar	5810.	8900.11	{8840., 8969.}	53.1861
26	R0	{1.4159, 4.93858}	{2.43135, 11.3148}	{{2.38758, 11.1331}, {2.45095, 11.4067}}	{71.7178, 129.11}	inf	48.	165.821	{55., 334.}	246.517	0.439395	newly	55.	82.8209	{28., 169.}	78.8602	0.238724	Reff	0.683172	0.298791	{0.269359, 0.32304}	56.2641	ar	5255.	8843.1	{8742., 8966.}	68.2798
27	R0	{1.57465, 7.05414}	{2.96307, 14.3219}	{{2.96307, 14.3219}, {2.96307, 14.3219}}	{88.1732, 103.028}	inf	116.	371.629	{180., 698.}	220.37	0.438221	newly	88.	149.887	{71., 277.}	77.4377	0.197755	Reff	0.599784	0.211453	{0.186673, 0.239712}	64.7451	ar	6359.	9382.58	{9331., 9427.}	47.5481
28	R0	{1.41422, 4.62402}	{2.45839, 11.6881}	{{2.45839, 11.6881}, {2.45839, 11.6881}}	{73.8336, 152.769}	inf	11.	229.705	{82., 360.}	1988.23	1.21163	newly	15.	113.	{41., 175.}	653.333	0.765255	Reff	0.643046	0.302725	{0.273619, 0.329178}	52.9233	ar	5485.	8870.92	{8797., 8938.}	61.7305
29	R0	{1.41901, 3.63017}	{2.52681, 9.219}	{{2.52681, 9.219}, {2.52681, 9.219}}	{78.0685, 153.955}	inf	5.	61.1587	{22., 110.}	1123.17	0.975559	newly	11.	35.5873	{12., 63.}	224.675	0.400985	Reff	0.571577	0.266895	{0.246869, 0.285277}	53.3055	ar	5979.	8966.44	{8904., 9041.}	49.9656
30	R0	{1.60719, 4.72984}	{2.92237, 11.8912}	{{2.91946, 11.8546}, {2.91946, 11.8546}}	{81.831, 151.408}	inf	1.	148.492	{71., 243.}	14 749.2	2.10041	newly	3.	69.8983	{31., 117.}	2229.94	1.29636	Reff	0.566374	0.200594	{0.184802, 0.223047}	64.5828	ar	6479.	9351.31	{9293., 9401.}	44.3325
31	R0	{1.38175, 6.26468}	{2.36114, 11.6557}	{{2.36114, 11.6557}, {2.36114, 11.6557}}	{70.8804, 86.0542}	inf	50.	206.927	{86., 379.}	313.855	0.490314	newly	41.	102.018	{40., 180.}	153.792	0.289321	Reff	0.648593	0.326052	{0.289712, 0.344726}	49.7572	ar	5397.	8725.85	{8638., 8820.}	61.6797
32	R0	{1.4947, 8.8847}	{2.24606, 16.668}	{{2.24606, 16.668}, {2.24606, 16.668}}	{50.2683, 87.6034}	inf	106.	348.825	{126., 662.}	231.96	0.436095	newly	69.	132.754	{47., 257.}	107.958	0.26717	Reff	0.721193	0.381846	{0.336235, 0.442698}	48.1792	ar	5407.	8526.33	{8436., 8612.}	57.6906
33	R0	{1.55891, 8.28978}	{2.42205, 19.4475}	{{2.42205, 19.4475}, {2.42205, 19.4475}}	{55.3682, 134.596}	inf	118.	577.621	{232., 1053.}	389.509	0.598711	newly	80.	188.69	{71., 335.}	141.853	0.305341	Reff	0.628553	0.388576	{0.30203, 0.426523}	46.7173	ar	6239.	8811.98	{8743., 8883.}	41.2403
34	R0	{1.60764, 10.4729}	{2.46477, 18.9032}	{{2.46477, 18.9032}, {2.46477, 18.9032}}	{53.316, 80.4963}	inf	168.	533.85	{220., 884.}	217.768	0.431494	newly	102.	178.65	{71., 309.}	87.9902	0.228794	Reff	0.600936	0.363085	{0.28616, 0.416546}	47.2756	ar	6495.	8883.57	{8780., 8947.}	36.7755
35	R0	{1.48527, 5.74485}	{2.67403, 14.2283}	{{2.59907, 13.5234}, {2.76203, 15.0558}}	{80.0367, 147.671}	inf	59.	248.2	{92., 368.}	320.782	0.520632	newly	58.	101.631	{41., 154.}	91.9363	0.229077	Reff	0.640597	0.25614	{0.226486, 0.282519}	60.0154	ar	5811.	9128.11	{8999., 9247.}	57.0833
36	R0	{1.61457, 9.93364}	{2.64373, 19.7855}	{{2.64373, 19.7855}, {2.64373, 19.7855}}	{63.742, 99.1767}	inf	204.	744.254	{365., 1308.}	264.83	0.502579	newly	131.	234.508	{111., 420.}	86.1626	0.22443	Reff	0.638401	0.325875	{0.26781, 0.402111}	49.169	ar	6338.	9088.49	{9032., 9142.}	43.3968
37	R0	{1.47831, 7.08139}	{2.56096, 15.0621}	{{2.55942, 15.0551}, {2.55942, 15.0551}}	{73.2358, 112.7}	inf	93.	503.516	{220., 869.}	441.415	0.652691	newly	79.	205.371	{86., 365.}	161.147	0.333589	Reff	0.683866	0.315348	{0.267971, 0.351408}	54.3306	ar	5530.	8999.58	{8925., 9073.}	62.7411
38	R0	{1.73445, 9.8858}	{2.98125, 18.4749}	{{2.98125, 18.4749}, {2.98125, 18.4749}}	{71.8845, 86.8832}	inf	168.	699.113	{336., 1080.}	316.139	0.559346	newly	85.	230.21	{115., 360.}	170.835	0.371806	Reff	0.583642	0.243054	{0.204514, 0.27666}	58.549	ar	6842.	9388.66	{9338., 9439.}	37.221
39	R0	{1.66621, 11.4824}	{2.64373, 19.7855}	{{2.64373, 19.7855}, {2.64373, 19.7855}}	{58.6673, 72.3115}	inf	221.	744.254	{365., 1308.}	236.767	0.467817	newly	130.	234.508	{111., 420.}	87.2772	0.226384	Reff	0.657653	0.325875	{0.26781, 0.402111}	50.5641	ar	6473.	9088.49	{9032., 9142.}	40.4062
40	R0	{1.60694, 9.95741}	{2.64373, 19.7855}	{{2.64373, 19.7855}, {2.64373, 19.7855}}	{64.5195, 98.7013}	inf	179.	591.206	{296., 1053.}	230.283	0.453122	newly	116.	187.54	{84., 363.}	72.6464	0.193452	Reff	0.592157	0.304339	{0.260407, 0.367214}	48.605	ar	6625.	9088.49	{9032., 9142.}	37.1848
41	R0	{1.55128, 4.71911}	{2.99356, 12.6328}	{{2.99519, 12.6436}, {2.99519, 12.6436}}	{92.9733, 167.695}	inf	16.	214.367	{88., 327.}	1239.79	1.0375	newly	16.	96.4333	{37., 144.}	502.708	0.681977	Reff	0.605309	0.192849	{0.173122, 0.212059}	68.1404	ar	6141.	9406.3	{9353., 9456.}	53.1721
42	R0	{1.60063, 6.81118}	{2.46477, 18.9032}	{{2.46477, 18.9032}, {2.46477, 18.9032}}	{53.9875, 177.532}	inf	61.	658.1	{272., 1078.}	978.852	0.971548	newly	44.	221.633	{91., 370.}	403.712	0.632258	Reff	0.565983	0.387088	{0.294294, 0.453518}	42.4414	ar	6528.	8883.57	{8780., 8947.}	36.084
43	R0	{1.56986, 5.44652}	{2.8186, 12.0293}	{{2.8186, 12.0293}, {2.8186, 12.0293}}	{79.5447, 120.862}	inf	36.	138.283	{61., 259.}	284.12	0.500261	newly	40.	67.05	{33., 129.}	77.125	0.193626	Reff	0.586343	0.215078	{0.195047, 0.233098}	63.3187	ar	6312.	9275.2	{9219., 9322.}	46.9455
44	R0	{1.47523, 8.07785}	{2.5963, 18.2333}	{{2.59755, 18.2464}, {2.59755, 18.2464}}	{75.9931, 125.719}	inf	154.	680.397	{281., 1483.}	341.816	0.556625	newly	118.	234.524	{99., 507.}	106.605	0.242976	Reff	0.674918	0.333696	{0.263132, 0.430414}	52.6813	ar	5692.	9049.83	{8987., 9121.}	58.992
45	R0	{1.6417, 7.69429}	{2.80057, 16.2348}	{{2.80057, 16.2348}, {2.80057, 16.2348}}	{70.5896, 110.998}	inf	70.	419.918	{227., 605.}	499.883	0.718266	newly	45.	155.525	{74., 227.}	245.61	0.480143	Reff	0.547999	0.248259	{0.223766, 0.274456}	54.6972	ar	6735.	9244.21	{9201., 9296.}	37.2563
46	R0	{1.38865, 5.83473}	{2.29055, 13.7413}	{{2.29055, 13.7413}, {2.29055, 13.7413}}	{64.948, 135.509}	inf	81.	442.455	{166., 864.}	446.24	0.649244	newly	88.	197.836	{65., 380.}	134.153	0.30075	Reff	0.6778	0.3838	{0.326861, 0.430165}	46.1822	ar	5233.	8615.65	{8528., 8694.}	64.6408
47	R0	{1.45289, 6.87903}	{2.55942, 15.0551}	{{2.55942, 15.0551}, {2.55942, 15.0551}}	{76.1606, 118.855}	inf	113.	505.242	{230., 869.}	347.117	0.571134	newly	105.	206.145	{90., 365.}	103.456	0.243565	Reff	0.690704	0.315572	{0.267971, 0.351408}	54.7183	ar	5379.	8999.02	{8925., 9073.}	67.2991
48	R0	{1.69416, 9.4831}	{2.64373, 19.7855}	{{2.64373, 19.7855}, {2.64373, 19.7855}}	{56.0496, 108.64}	inf	191.	925.825	{470., 1599.}	384.725	0.634441	newly	108.	296.571	{149., 508.}	174.603	0.384254	Reff	0.561783	0.356278	{0.279442, 0.45525}	39.687	ar	6925.	9088.49	{9032., 9142.}	31.2418
49	R0	{1.47843, 7.16286}	{2.51625, 15.1621}	{{2.51743, 15.191}, {2.51743, 15.191}}	{70.1977, 111.677}	inf	84.	437.889	{192., 856.}	421.296	0.62535	newly	57.	171.127	{72., 327.}	200.613	0.382004	Reff	0.694419	0.313199	{0.271637, 0.346147}	54.8977	ar	5449.	8953.33	{8875., 9007.}	64.3115
50	R0	{1.68843, 5.72427}	{2.93653, 10.872}	{{2.93653, 10.872}, {2.93653, 10.872}}	{73.9207, 89.9281}	inf	27.	70.0984	{36., 107.}	159.866	0.351391	newly	30.	35.1148	{17., 55.}	48.9617	0.174946	Reff	0.541311	0.190711	{0.172081, 0.207319}	64.7687	ar	6800.	9367.05	{9316., 9430.}	37.7507
